# Adverse Effects of Subchronic Dose of Aspirin on Reproductive Profile of Male Rats

**DOI:** 10.1155/2016/6585430

**Published:** 2016-04-12

**Authors:** Archana Vyas, Heera Ram, Ashok Purohit, Rameshwar Jatwa

**Affiliations:** ^1^Department of Zoology, Jai Narain Vyas University, Jodhpur, Rajasthan 342001, India; ^2^Molecular Medicine and Toxicology Lab, School of Life Sciences, Devi Ahilya University, Indore, Madhya Pradesh 452001, India

## Abstract

Aspirin (acetylsalicylic acid) is widely used for cardiovascular prophylaxis and as anti-inflammatory pharmaceutical. An investigation was carried out to evaluate the influence of subchronic dose of aspirin on reproductive profile of male rats, if any. Experimental animals were divided into three groups: control and aspirin subchronic dose of 12.5 mg/kg for 30 days and 60 days, respectively, while alterations in sperm dynamics, testicular histopathological and planimetric investigations, body and organs weights, lipid profiles, and hematology were performed as per aimed objectives. Subchronic dose of aspirin reduced sperm density, count, and mobility in cauda epididymis and testis; histopathology and developing primary spermatogonial cells (primary spermatogonia, secondary spermatogonia, and mature spermatocyte) count were also significantly decreased in rats. Hematological investigations revealed hemopoietic abnormalities in 60-day-treated animals along with dysfunctions in hepatic and renal functions. The findings of the present study revealed that administration with subchronic dose of aspirin to male rats resulted in altered reproductive profiles and serum biochemistry.

## 1. Introduction

Aspirin (acetylsalicylic acid) is a nonsteroidal anti-inflammatory drug (NSAID) used in various pathological conditions for its anti-inflammatory, antipyretic, and analgesic benefits [1, 2]. Investigations on aspirin and its underlying mechanism exposed new arena of knowledge, namely, prostaglandin synthesis and platelet inhibition and allowed additional development of efficient antiplatelet agents and anti-inflammatory medications [3]. In the present scenario, with increasing incidence of noncommunicable diseases, aspirin has gained a significant attention not only as an analgesic but also as a cardioprotective agent [4]. On the other hand, reports are there in the literature suggesting morbidity and mortality associated with adverse effects of aspirin. Furthermore, long-term therapeutic use of aspirin is associated with the incidences of gastrointestinal (GI) ulcerations, nephrotoxicity, hepatotoxicity, and even renal cell cancers [5]. The antiplatelet effect of aspirin has been attributed to coronary artery disease, pregnancy complications, and preeclampsia in angiotensin-sensitive primigravida [5–7]. Whereas aspirin treatment causes an increased risk of cerebral microbleeds, tinnitus in children, and Reye's syndrome when given to children or adolescents to treat fever or illnesses, it alters estrogen and progesterone biosynthesis upon chronic administration [8–10].

Interestingly, aspirin-induced inhibition of prostaglandins synthesis resulted in altered cholesterol metabolism and androgen biosynthesis [10]. However, effect of subchronic aspirin administration on male reproductive profile was not well elucidated till date. Therefore, the present study was designed to ravel out the influence of aspirin subchronic dose on male reproductive profile and serum variables of rats.

## 2. Materials and Methods

### 2.1. Experimental Animals

Colony bred adult healthy male albino rats weighing 200–235 g were used for present research. All animals were proven fertile and were obtained from the Indian Veterinary Research Institute (IVRI), Bareilly, India. They were housed in a standard light (14 h light : 10 h dark cycle), controlled room temperature (23 ± 2°C), with the provision of laboratory feed and water ad libitum. The animals were acclimatized for a week before conducting the experiment and care of the laboratory animals was taken as per the guidelines laid down by the Committee for the Purpose of Control and Supervision on Experiments on Animals (CPCSEA), government of India, New Delhi (reg. number: 1646/GO/ERe/S/12/CPCSEA).

### 2.2. Drug and Dose Regime

Aspirin (acetylsalicylic acid) tablets (Ecosprin-75®) were purchased from the local registered medical store. Aspirin dose regime was given at low dose of 12.5 mg/kg body weight for 30 and 60 days as per protocol.

### 2.3. Experimental Design and Preparation of Serum and Tissue Samples

Twenty-one adult healthy rats were randomly divided into three groups of seven each. While animals of group 1 received vehicle, distilled water served as control and group 2 and 3 animals received aspirin (12.5 mg/kg) by oral gavage for 30 days and 60 days, respectively. On the day of termination, day 31 (group 2) and day 61 (group 3), overnight fasted animals were autopsied after exposing them to mild ether anesthesia. Blood from each animal was collected by cardiac puncture method and serum was isolated and stored at –20°C until assayed for glucose, lipid profiles, and other parameters.

### 2.4. Estimation of Total Cholesterol

Total cholesterol concentration in serum was determined by an enzymatic method using commercial available test kit as was routinely done in our laboratory [11, 12]. In brief, cholesterol esters are enzymatically hydrolyzed by cholesterol esterase to cholesterol and free fatty acids. The hydrogen peroxide combines with 4-aminoantipyrine to form a chromophore (quinoneimine dye) which was monitored on a spectrophotometer at 505 nm.

### 2.5. Triglyceride Assay

Circulating triglyceride was determined by an enzymatic method using a commercial available triglyceride test kit method. Triglycerides are enzymatically hydrolyzed by lipase to free acids and glycerol. Color reaction is catalyzed by peroxidase; H_2_O_2_ reacts with 4-aminoantipyrine (4AAP) and 4-chlorophenol to produce a red colored dye [12, 13].

### 2.6. Fasting Glucose Estimation

Circulating fasting serum glucose level was measured by a standard method as routinely followed in our laboratory. In brief, glucose is oxidized by glucose oxidase/peroxidase enzyme to yield gluconic acid and hydrogen peroxide. The enzyme peroxidase catalyzes the oxidative coupling of 4-aminoantipyrine with phenol to yield a colored quinoneimine complex. The absorbance is proportional to the concentration of glucose in the sample [14].

### 2.7. Analysis of Serum Urea

Urea level in serum was measured by standard diacetyl monoxime method. The enzyme methodology employed as urea is hydrolyzed in the presence of water and urease to produce ammonia and carbon dioxide. The reaction is monitored by measuring the rate of decrease in absorbance at 340 nm as NADH is converted to NAD [15].

### 2.8. Estimation of Creatinine

Serum creatinine level was measured by kit method as described earlier. Creatinine reacts with alkaline picrate to produce an orange-yellow color. The absorbance of the orange-yellow color formed is directly proportional to creatinine concentration and is measured on a spectrophotometer at 490–510 nm [16].

### 2.9. Analysis of SGOT and SGPT Activities

Measurement of SGOT and SGPT was done by enzymatic methods. In brief, series of reactions involved in the assay for SGOT as the sample catalyzes the transfer of the amino group from L-aspartate to 2-oxoglutarate forming oxaloacetate and L-glutamate. The reaction is monitored by measuring the rate of decrease in absorbance at 340 nm due to the oxidation of NADH to NAD.

Similarly, SGPT catalyzes deamination from alanine to 2-oxoglutarate yielding pyruvate and L-glutamate. Pyruvate is reduced to lactate by LDH present in the reagent with the simultaneous oxidation of NADH to NAD. The reaction is monitored by absorbance at 340 nm on a UV-Vis spectrophotometer [17].

### 2.10. Quantitative Estimation of Total Protein

Total serum protein level was estimated by using the method of Lowry et al. [18], as routinely performed in our laboratory. The peptide bonds of protein react with Cu^+2^ ions in alkaline conditions to form a blue-violet ion complex. The color formed is proportional to the protein concentration and is measured at 660 nm on a UV-Vis spectrophotometer [18].

### 2.11. Hematological Analysis

Blood samples were collected by direct cardiac puncture method and hematological assessments were performed following standard protocols. The hematological measurements included red blood corpuscles (RBC) count, white blood corpuscle (WBC) count, platelet counts, hemoglobin (Hb) concentration, and hematocrit (HCT) by Wintrobe's methods [12, 19].

### 2.12. Sperm Dynamics Analysis

The sperm dynamics analysis was performed following standard method, as routinely done in our laboratory. In brief, the cauda epididymis from each animal was chopped into phosphate buffered saline (0.1 M, pH 7.4) and the following observations were made:

- Total number of sperms.
- Total number of motile sperms.
- Normal and abnormal sperm counts.
- Sperms densities in testis and cauda.

The total sperm count was calculated by using Neubauer's haemocytometer. The accuracy of sperm count was increased; the epididymis plasma was diluted with a spermicidal solution, prepared by dissolving 5 g of NaHCO3 and 1 mL of 40% formaldehyde in 100 mL of normal saline. A twenty times dilution was made by using WBC pipette, which was thoroughly mixed and one drop was added to both sides of Neubauer's haemocytometer. The sperms were allowed to settle down in the haemocytometer by keeping them in a humid chamber for one hour. The sperm count was done in 5 major squares designated as *E*1, *E*2, *E*3, *E*4, and a central *E*5. Each square is 1 mm long, 1 mm wide, and 0.1 mm high. The total volume represented by each major square *E* is thus 0.1 mm^3^ or 10^−4^ mm. The total number of sperms was counted in all the major squares and calculated as follows:

(1) Total no. of sperms/mL plasma=Total no. of sperms per square XTotal volume per square 10−4×dilution factor 20.

Similarly, the total number of motile sperms was calculated by using PBGS (phosphate buffered glucose saline) instead of spermicidal solution [20, 21].

### 2.13. Testicular Histopathological and Planimetric Investigations

After exsanguinations, testis tissues were removed, quickly freed from blood clots, washed thoroughly with phosphate buffered saline (0.1 M, pH 7.4), and used for fixation. Fixed tissues were prepared under paraffin embedding, stained with hematoxylin-eosin (H&E), and examined at 5 *μ*M thick sections. Microscopic observations were made at 100x and 400x magnifications with proper resolution of testicular histoarchitectural alterations. Consequently, planimetric study was performed at 8 × 4 magnifications for germ cell population dynamics by camera lucida [22].

## 3. Results

### 3.1. Effect on Body and Organs Weight

The body weight of the rats was not significantly altered in 30-day- and 60-day-treated groups. Animals in groups 2 and 3 exhibited a significant decrease in the weight of testis and cauda epididymis (*P* ≤ 0.001 for both). Similarly, weights of ventral prostate and seminal vesicle of the animals of 30-day and 60-day treatment groups also showed significant decrease (*P* ≤ 0.001 for all), whereas there were no significant changes observed in the weights of liver, kidney, and body weights after 30 days or 60 days of treatment, as compared to vehicle treated controls (Table 1).

### 3.2. Effect on Sperm Dynamics

Analysis of sperm dynamics, namely, total sperm count, total number of motile sperms, sperm density, and abnormal sperm of the cauda epididymis and testis was carried out in the control and animals of 30-day- and 60-day-treated groups.

Aspirin administration for 30 days to rats resulted in significant decrease (*P* ≤ 0.001 for all) in sperm dynamics parameters of both groups (Table 2). Total sperm counts in testis and cauda significantly decreased (*P* ≤ 0.001 for both) in 30-day- and 60-day-treated group in a gradual manner. Concurrently, the sperm density in cauda and testis was reduced (*P* ≤ 0.001 for both) in both the groups who received subchronic dose of aspirin for 30 days and 60 days. Sperm mortality also increased following aspirin treatment for 30 days and 60 days (*P* ≤ 0.001 for both) as compared to vehicle control group (Figures 2(a) and 2(b)).

### 3.3. Serum Biochemistry

The results of serum biochemistry are depicted in Table 3. The concentrations of serum total protein, cholesterol, triglyceride, and glucose were not altered significantly following aspirin administration compared to controls, while serum SGOT and SGPT activities and creatinine and urea levels were increased in both the groups who received subchronic aspirin treatments for 30 and 60 days (Table 2).

### 3.4. Hematological Study

**Figure 1:**
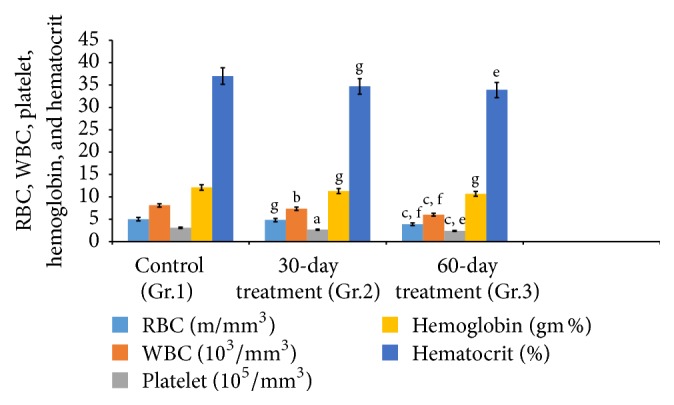
Effect of aspirin subchronic dose administration on RBC, WBC, platelets, hemoglobin, and hematocrit of rats. Data are means ± SEM (*n* = 7). ^a^ *P* ≤ 0.05: ^b^ *P* ≤ 0.01, ^c^ *P* ≤ 0.001 as compared to the respective control values; ^e^ *P* ≤ 0.01 and ^f^ *P* ≤ 0.001 as compared to the respective values of the 30-day-treated groups and ^g^nonsignificant.

**Figure 2:**
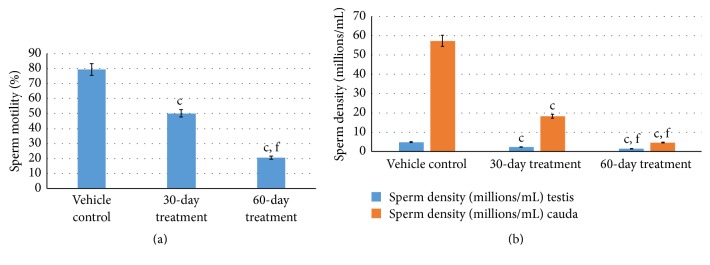
(a) Effect of aspirin subchronic dose administration on sperm motility of treated rats. Data are means ± SEM (*n* = 7). ^c^ *P* ≤ 0.001 as compared to the respective control values; ^f^ *P* ≤ 0.001 as compared to the respective values of the 30-day-treated group. (b) Effects of aspirin subchronic dose administration on sperm density on treated rats. Data are means ± SEM (*n* = 7). ^c^ *P* ≤ 0.001 as compared to the respective control values; ^f^ *P* ≤ 0.001 as compared to the respective values of the 30-day-treated group.

Aspirin treatments influenced the hematology of the rats. Hematological parameters, namely, Hb (hemoglobin) concentrations, RBC count, WBC count, and HCT (hematocrit) of groups 2 and 3 exhibited significant alterations as compared to controls (Figure 1).

### 3.5. Testicular Histopathological and Planimetric Investigations

**Figure 3:**
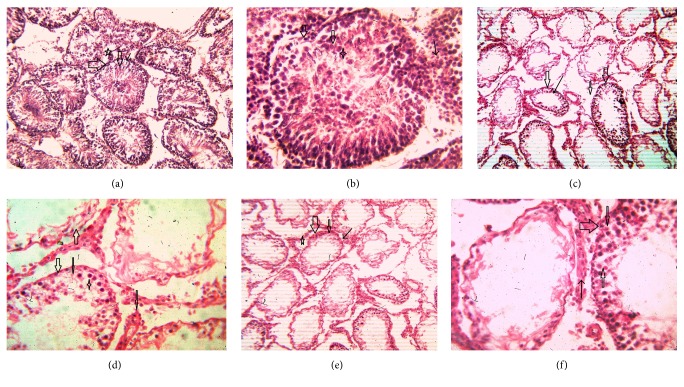
(a) Testis histoarchitecture of vehicle control rats (100x HE). Seminiferous tubules consisting of germ epithelium cells, various stages of spermatogonia, and spermatids as normal histoarchitecture of testis (thick arrow). Sertoli cells also exist as supporting and nutritioning cells (→). Interstitial cells or Leydig's cells existing between interseminiferous tubular spaces along with proper framing of connective tissues (star sign). (b) Testis histoarchitecture of vehicle rats (400x HE). At higher magnification, a particular seminiferous showing germ epithelium cells, sertoli cells, and various stages of spermatogonia and spermatids (thick arrow, narrow arrow, and star). (c) Testis histoarchitecture of 30-day-aspirin-subchronic-dose-treated rats (100x HE). The aspirin treatment showing cytological and nuclear degenerative changes in seminiferous tubules (thick arrow) and surrounding tissues as abnormal histoarchitecture (middle sized arrow and thin arrow). (d) Testis histoarchitecture of 30-day-aspirin-subchronic-dose-treated rats (400x HE). A 30-day treatment of aspirin caused abnormal histoarchitecture on particular seminiferous tubule as cytological toxicity by decreased numbers of various stages of spermatogonia (thin arrow). Interstitial cells showing in normal conditions as shown by middle sized arrow. Star showing degenerating stages of spermatogonia. (e) Testis histoarchitecture of 60-day-aspirin-subchronic-dose-treated rats (100x HE). Long-term aspirin treatment caused abnormal histoarchitecture as degenerations and reduced numbers of various stages of spermatogonia (middle sized arrow) and peripheral tissues (star). Thick arrow showing shrinkage in seminiferous tubules. (f) Testis histoarchitecture of 60-day-aspirin-subchronic-dose-treated rats (100x HE). 60-day aspirin treatment caused degenerative changes in particular seminiferous tubule as showing reduced numbers of various stages of spermatogonia (indicated by middle sized arrow), sertoli cells (first middle arrow), and without affecting Leydig's cells (thin arrow).

Histoarchitecture of control group testis showed normal testicular architecture with an orderly arrangement of spermatogenic developing cells and interstitial and sertoli cells with connective tissues. The spermatogonia, that is, primary, secondary, and sertoli cells were rested on the basement membrane of the seminiferous tubules. Leydig's cells with large and acidophilic cytoplasm were located in the interstitial tissue among seminiferous tubules (Figures 3(a)-3(b)), while treatment with subchronic dose of aspirin for 30 days or 60 days resulted in varying degrees of abnormalities in testis histology. The primary spermatogonia, secondary spermatogonia, spermatocytes, and Leydig's cells exhibited significant reduction (*P* ≤ 0.001 for all) following administration of aspirin for 30 or 60 days (Figures 3(c)-3(d) and Figures 3(e)-3(f)). Concurrently, population of developing spermatogonial cells and fibroblast cells were reduced (*P* ≤ 0.001 for all) in aspirin treated groups as compared to vehicle control (Table 3).

## 4. Discussion

Aspirin is one of most widely used anti-inflammatory drugs with proven cardiovascular benefits [23]. Nonsteroidal anti-inflammatory drugs (NSAIDs) are suggested for analgesic and anti-inflammatory activities to manage oncologic and neurologic diseases in human and veterinary subjects. Despite putting efforts in improving the safety, efficacy, and potency of NSAIDs, adverse effects such as gastrointestinal irritation, renal and hepatic toxicity, interference with hemostasis, and reproductive problems still persist [24]. The findings of the present study clearly reveal that subchronic administration (for 30 or 60 days) of aspirin to male rats caused reproductive abnormalities and liver toxicity. However, treatment with aspirin was found to be safe with reference to organs and body weight, except testis, epididymis, seminal vesicle, and ventral prostate. These alterations might be conducted through indirect involvement of inhibition of androgens biosynthesis [23, 24]. It is well understood that weight, size, and secretary function of testes, epididymis, seminal vesicles, ventral prostate, and vasa differentia are closely regulated by androgens hormones. However, the process of spermatogenesis and the accessory reproductive organs function are dependent on androgen activity [24, 25].

Interestingly, aspirin administration to normal rats resulted in hypercholesterolemia. The increased levels of cholesterol and triglyceride in aspirin treated groups might be due to decreased androgen production, which resulted in accumulation of cholesterol in testes. The impaired sperm dynamics, including spermatogenesis, could be an outcome of aspirin-induced alteration in cholesterol metabolism in testis [26].

Interestingly, subchronic aspirin treatment resulted in reduced sperm count and density; on the other hand, it enhanced sperm motility. Androgens are essential for survival and motility of spermatozoa in the epididymis and cauda region appears to be the most favorable site for the same. Aspirin-induced reduction in sperm dynamics might be an outcome of depressed levels of androgens. Interestingly, subchronic aspirin administration influenced androgen dependent parameters including that of reduced sperm count, motility, and density. The observed reduced sperm activity profile might be an outcome of androgen depletion at target level, particularly in the cauda epididymis, thereby affecting physiological maturation of the sperm [26–28]. In the literature, reports are suggesting that reproductive toxicant which affects sperm motility would in turn influence spermatozoa indirectly through disruption of epididymis epithelial cell function and/or by acting directly on the spermatozoa by affecting their enzymes [26, 27]. It is speculated that aspirin might have caused reproductive toxicity in male rats through this mechanism, as evidenced by the reduction in androgen dependent parameters.

On the other hand, aspirin treatment for 60 days elevated serum levels of urea, uric acid, creatinine, and the activities of SGOT and SGPT, reflecting the toxic effects on subchronic administration. Adverse effects following aspirin administration in normal rats might be an outcome of NSAIDs induced inhibition of prostaglandin synthesis which leads to renal vasoconstriction and decreased renal perfusion which is responsible for acute renal abnormalities [28–30]. Aspirin-induced liver toxicity is obvious, as liver is the major target organ for drug metabolism and hepatic biotransformation reactions are known to induce apoptosis of hepatocytes [31]. Furthermore, aspirin-induced liver toxicity could be an outcome of idiosyncratic metabolic reaction due to aberrant metabolism of the drug where accumulation of toxic metabolites in hepatocytes binds to cell proteins and leads to abnormalities [30, 31]. Observations made on total protein levels are in accordance with this fact. The alterations in the level of serum proteins might be an outcome of both hepatic and renal damage, where it causes alteration in a number of enzymes and could also disturb protein synthesis in hepatocytes [31]. Moreover, decreased serum protein levels could be attributed to a reduced hepatic DNA and RNA synthesis which in turn hampered utilization of free amino acids for protein synthesis [32, 33]. In the literature, aspirin-induced hematological disorders are well documented, and the results of the present study are in accordance with this which is suggesting aspirin-induced malfunctions in hemopoiesis of male rats [34, 35].

In conclusion, subchronic administration (for 30 or 60 days) of aspirin resulted in reduced sperm density, count, and mobility in cauda epididymis and testis as well as developing spermatogonial cells count, reflecting the toxic nature in rats. Findings made on the hepatic and renal profiles further reveal toxic nature of the aspirin when administered to rats subchronically.

## Figures and Tables

**Table 1:** Adverse effects of subchronic dose of aspirin on body and organs weight of male rats.

Treatment groups	Body weight (g)		Testes	Epididymis	Seminal vesicle	Ventral prostate	Heart	Kidney	Liver
	Initial	Final	mg/100 g body weight						g/100 g BW
Control (Gr. 1)	211.3 ± 18.2	227 ± 19.51	1209 ± 72.5	560 ± 15.6	618.6 ± 18.6	279.7 ± 7.4	315.5 ± 16.2	595 ± 25.3	2.6 ± 0.12
30-day-treated group (Gr. 2)	220 ± 20.12^g^	228 ± 19.12^g^	634 ± 30.16^c^	368 ± 13.6^c^	273 ± 10.12^c^	126 ± 5.76^c^	363 ± 11.12^g^	608 ± 21.36^g^	2.8 ± 0.61^g^
60-day-treated group (Gr. 3)	230 ± 22.1^g^	237 ± 21.67^g^	724 ± 49.79^c^	396 ± 16.17^c^	319 ± 13.79^c^	136 ± 6.12^c^	329 ± 12.63^g^	642 ± 20.12^g^	2.9 ± 0.36^g^

Data are means ± SEM (*n* = 7). ^c^ *P* ≤ 0.001 compared to the respective control values; ^g^nonsignificant.

**Table 2:** Adverse effects of subchronic dose of aspirin on testicular cell population dynamics of rats.

Groups	Germinal cell types				Interstitial cell types			
	Spermatogonia	Primary spermatocyte	Secondary spermatocyte	Spermatids	Fibroblast	Immature Leydig's cell	Mature Leydig's cell	Degenerating cell
Vehicle control	26.12 ± 0.76	20.25 ± 0.86	65.16 ± 3.16	160.19 ± 5.16	59.01 ± 1.32	51.33 ± 2.16	73.16 ± 1.06	16.36 ± 1.37
30-day treatment	19.61 ± 1.73^b^	12.25 ± 0.62^c^	12.16 ± 1.26^c^	5.19 ± 0.76^c^	55.07 ± 2.31	30.33 ± 3.01^c^	32.16 ± 3.05^c^	70.16 ± 1.31^c^
60-day treatment	5.23 ± 0.36^c,f^	2.25 ± 0.26^c,f^	1.56 ± 0.96^c,f^	2.19 ± 5.16^c,f^	47.01 ± 2.32^b^	21.33 ± 2.67^c^	19.01 ± 1.06^c,f^	81.12 ± 2.16^c,f^

Data are means ± SEM (*n* = 7). ^b^ *P* ≤ 0.01; ^c^ *P* ≤ 0.001 compared to the respective control values and ^f^ *P* ≤ 0.001 compared to the respective values of the 30-day-treated group.

**Table 3:** Effects of subchronic dose of aspirin on serum total cholesterol, triglyceride, glucose, urea, creatinine, and protein and activities of SGOT and SGPT of male rats.

Groups	Cholesterol (mg/dL)	Glucose (mg/dL)	Triglyceride (mg/dL)	Urea (mg/dL)	Creatinine (mg/dL)	SGOT (U/L)	SGPT (U/L)	Protein (mg/dL)
Control (Gr. 1)	104.1 ± 3.34	97 ± 5	90.21 ± 5.2	21.12 ± 2.16	1.01 ± 0.12	40 ± 3.2	40 ± 2.6	5.52 ± 1.02
30-day-treated group (Gr. 2)	121 ± 7.36^g^	103 ± 3.34^g^	102.02 ± 2.1^g^	30.5 ± 2.76^g^	1.65 ± 0.04^a^	210 ± 5.26^c^	93 ± 2.36^c^	7.13 ± 1.63^g^
60-day-treated group (Gr. 3)	111 ± 5.23^g^	107 ± 4.32^g^	97.11 ± 3.1^g^	39 ± 1.96^g^	1.86 ± 0.043^b^	251 ± 6.16^c,f^	190 ± 2.67^c,f^	7.87 ± 1.24^g^

Data are means ± SEM (*n* = 7). ^a^ *P* ≤ 0.05; ^b^ *P* ≤ 0.01; ^c^ *P* ≤ 0.001 compared to the respective control values; ^f^ *P* ≤ 0.001 compared to the respective values of the 30-day-treated group and ^g^nonsignificant.
